# 2020 Syndemic: Convergence of COVID-19, Gender-Based Violence, and Racism Pandemics

**DOI:** 10.1007/s40615-021-01146-w

**Published:** 2021-10-14

**Authors:** Nazilla Khanlou, Luz Maria Vazquez, Soheila Pashang, Jennifer A. Connolly, Farah Ahmad, Andrew Ssawe

**Affiliations:** 1 Faculty of Health, York University, 4700 Keele Street, ON M3J 1P3 Toronto, Canada; 2 Faculty of Social and Community Services, Humber Institute of Technology and Advanced Learning, ON Toronto, Canada; 3 Newcomers, Families and Clinical Programs and Services, South Riverdale Community Health Centre, ON Toronto, Canada

**Keywords:** COVID-19, Racism, Gender-based violence, Mental health, Pandemics

## Abstract

**Objective:**

To conduct a rapid knowledge synthesis of literature on the social determinants of mental health of racialized women exposed to gender-based violence (GBV) during the COVID-19 pandemic.

**Methods:**

We adapted the Cochrane Rapid Reviews method and were guided by an equity lens in conducting rapid reviews on public health issues. Four electronic databases (Cochrane CENTRAL, Medline, ProQuest, and EBSCO), electronic news media, Google Scholar, and policy documents were searched for literature between January 2019 and October 2020 with no limitations for location. Fifty-five articles qualified for the review.

**Results:**

Health emergencies heighten gender inequalities in relation to income, employment, job security, and working conditions. Household stress and pandemic-related restrictions (social distancing, closure of services) increase women’s vulnerability to violence. Systemic racism and discrimination intensify health disparities.

**Conclusion:**

Racialized women are experiencing a *2020 Syndemic*: a convergence of COVID-19, GBV, and racism pandemics, placing their wellbeing at a disproportionate risk. GBV is a public health issue and gender-responsive COVID-19 programming is essential. Anti-racist and equity-promoting policies to GBV service provision and disaggregated data collection are required.

## Introduction

The COVID-19 pandemic has led to differential health, social, and economic impacts on populations, illuminating the prevailing inequalities that reinforce disadvantage for marginalized segments of the public [1, 2]. Among the disturbing outcomes of the pandemic is the global rise in gender-based violence (GBV) [3], including sexual assault and rape, experienced by women [4]. The United Nations has referred to this alarming societal problem as the “shadow pandemic” [3]. Fear, uncertainties, and stressors among the population during the pandemic can increase anger and aggression and further lead to victimization of women [5]. This is of concern given that in the 12 months previous to the COVID-19 pandemic, 243 million women and girls (aged 15–49) around the world were subjected to sexual and/or physical violence [5].

Gender-based violence is perpetrated against individuals based on their perceived gender, gender identity, or gender expression and may include a range of human rights violations, such as rape, domestic violence, sexual assault and harassment, trafficking of women and girls, and sexual abuse of children. Estimates show GBV impacts the mental, physical, and sexual health of an alarming 30–60% of women globally [6]. Violence against women contributes to high levels of morbidity and mortality [7]. The life-long impacts of GBV include psychological distress, anxiety disorders (post-traumatic stress disorder), depression, and substance use disorders [8]. Higher rates of past suicide attempts [7, 9] social exclusion, and isolation among women have also been reported [8]. Violence against women is rooted in discrimination and inequality that are sustained and reproduced by systems and structures as well as social norms.

Life free of violence is a fundamental human right and to achieve equality and nondiscrimination a lot more needs to be done to address GBV. This has become crucial under the pandemic with escalating GBV incidents [4]. Addressing this urgent public health and policy and practice need, our team conducted a *rapid knowledge synthesis* of the literature with a focus on racialized women. We looked at the racialized and gendered social determinants of mental health among women exposed to gender-based violence during the COVID-19 pandemic. We report on the literature review methods and synthesis findings in this paper. Overall, we found that racialized women are experiencing what we name as a *2020 Syndemic*: a convergence of COVID-19, gender-based violence, and racism pandemics, placing their wellbeing at a disproportionate risk. The term syndemic refers to two or more epidemics [10]. A syndemic lens illuminates how diseases are aggravated by socioeconomic, political, or environmental contexts, and how they interact, leading to synergistic vulnerability to diseases and social inequities [11].

### An Intersectional Approach to COVID-19 as a Syndemic

A syndemic framework integrates two processes: (i) disease concentration (clustering of multiple epidemics, which results from political economic contexts); and (ii) disease interaction (to explain how multiple epidemics exacerbate health effect in contexts of “adverse social conditions”) [12]. The concept emerged in the 1990s, coined by medical anthropologist Merrill Singer [13]. Singer et al. explain [14] that syndemics are most likely to emerge in the context of health and socioeconomic inequalities including poverty, stigmatization, stress, or structural violence, because these factors account for disease clustering and exposure, and also promote increased physical and behavioral vulnerability.

Horton [15] states that the COVID-19 is not a pandemic, but a syndemic, and that we need to look at the contexts of socioeconomic inequalities that are disproportionally impacting particular marginalized groups in our society. Horton emphasizes that viewing COVID-19 as a syndemic points out its underlining social origins [15]. A focus on the social dimensions allows us to better understand the factors which contribute to the formation of disease, its clustering, and its spread [16]. It also allows an understanding of conditions which further contribute to disease progression such as reducing an individual’s immune function and increasing susceptibility to disease [16]. A syndemic perspective then is a lens to explain why certain individuals, families, or communities are more vulnerable than others [10]. Violence is considered a dimension of syndemics and was first integrated into this approach in the analysis of substance abuse and HIV [17]. Researchers apply, for example, the concept of *social comorbidities* to refer to family and community violence, sexual assault, childhood sexual abuse, depression, and substance abuse, as behavioral and social phenomena aspects of violence against women [17].

While a good start to addressing compounding health risks, the epidemiologic lens needs to be coupled with critical perspectives to address complex social inequities and marginalization [15, 18]. In our studies on mental health support for racialized women at risk of gender-based violence, we apply an intersectionality-informed approach to analyzing the combined impacts of racism, sexism, and social inequities. An intersectionality lens, we believe, enables us to examine how gender intersects with the social determinants of health, leading to gendered health disparities pathways, and resulting in synergistic health disadvantage for certain segments of the population, including racialized women at risk of gender-based violence during COVID-19 pandemic’s response and recovery phases.

Intersectionality is an analytical approach for understanding and responding to the ways in which gender intersects with identity markers—including race, socioeconomic status, dis(ability), sexual orientation, migration status, ethnicity—and how these intersections contribute to unique experiences of privilege and oppression [19, 20] People live multiple, layered identities, derived from social relations, history, and the operation of structures of power [20]. Women’s experiences are influenced by social identities that are impacted by power/lessness, marginalization, and structural inequities. Therefore, how women address violence, the resources they have, the barriers they face to access the services they need, the resiliency they have to cope with adversity, is informed and influenced by the contextual intersections [20]. We can think of intersectionality as an approach for development and human rights work, and to promoting a social justice action agenda. It has been argued that the frameworks of intersectionality and syndemics “are rarely brought together” [18]. Through sharing findings of our rapid synthesis review, we hope to inform public health interventions addressing GBV, and to efforts mitigating the negative social determinants of mental health impacts of the COVID-19 syndemic on racialized women.

## Methods

We adapted the *Cochrane COVID Rapid Reviews Method* [21] in conducting rapid reviews on public health issues. Our methods were guided by the Centers for Disease Control and Prevention (CDCP) [22] *Equity Lens*, which considers the many health inequities (such as health care access and utilization, discrimination, education, income, wealth gaps, occupation, and housing) that put racial and ethnic minority groups at increased risk during the COVID-19 pandemic. The review included the following six steps. In *Step one*, we identified two research questions: (i) “What are the racialized and gendered social determinants of mental health among racialized women and girls with experiences of GBV?”; and (ii) “What are the emerging best practices/evidence of effectiveness of services or implementation for equity-informed mental health promotion and health care provision for this population during the current COVID-19 pandemic?”. In *Step two*, we set the *study selection criteria* (study inclusion criteria): PICO was applied: **P**opulation: women and/or girls 15 years and older at risk of GBV and experiencing GBV. **I**ntervention: published studies assessing/addressing GBV and mental health outcomes (including interventions/initiatives) during the COVID-19 pandemic. There were **no C**omparators. **O**utcomes: identified emerging guidelines. *Step three* involved a search strategy to identify relevant studies. Searches were conducted across four electronic databases (Cochrane CENTRAL, Medline, ProQuest, and EBSCO). We examined ongoing/unpublished studies through grey literature searching of websites, including electronic news media, Google Scholar, and policy documents, between the years 2019 and October 2020. The search strategy and keywords were developed by the research team members and approved by the principal investigator (first author) and a health sciences librarian (Table 1).

**Table 1:** Search terms

Key Search Terms	
1	women, woman, gender
2	violence, abuse, stress, domestic violence, intimate partner violence
3	health, mental health, wellbeing, well being
4	pandemic, COVID, coronavirus
5	migrant*, immigrant*, precarious status, racial*, race, Asia*, Latin*, Hispanic, Black, African American, Indigenous, aboriginal, native, ethnic minority, minority, ethnocultural

**Fig. 1:**
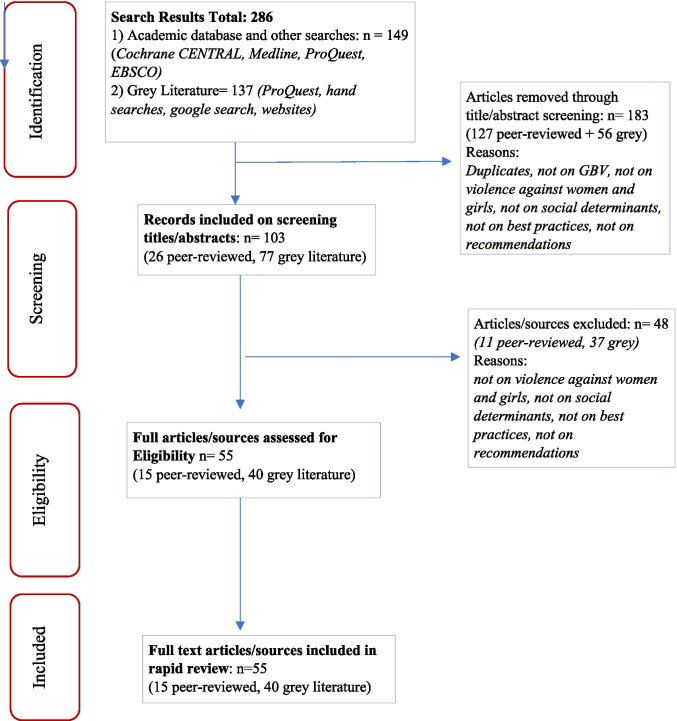
PRISMA diagram—rapid review search results

Data collection was completed in *Step four.* Key characteristics of selected studies/articles were recorded after reviewing 286 items including journal articles, book chapters, and grey literature. For the final review, *55 articles* met the eligibility criteria (15 peer-reviewed and 40 grey literature). Excluded sources included duplicates and material that did not focus on violence against women and girls, social determinants of health, best practices, or recommendations. Analysis and synthesis were completed in steps 5 and 6. In *Step five*, emerging review findings were organized along following the overall themes: (1) COVID-19 social determinants of health and impacts on women; (2) Gender-based violence risk factors during a pandemic; and (3) Racism, discrimination, and health outcomes. We interpreted the public health implications of the findings through an intersectionality-informed lens. In *Step six*, we considered applicability and transferability of the findings through application of a syndemic understanding of the themes and identification of multilevel interventions (Fig. 1).

## Results

The findings of our rapid synthesis address three significant and overlapping areas, as outlined in further detail in the following sections.

### COVID-19 Social Determinants of Health and Impacts on Women

Experiences from previous epidemics indicate that women’s burdens (physical, psychological, and time) increase during health emergencies [5]. In the context of the current COVID-19 pandemic, this is not the exception. Health emergencies heighten pervasive existing gender inequalities. Crises increase women’s vulnerabilities given they are more likely than men to work in informal and precarious minimum wage jobs [23], and as frontline providers [9], representing 70% of the health and social care workforce around the world [24]. Women engage in frontline health, social services, and cleaning professions, which place them at higher risk in the current pandemic. Depending on location, it is important to note the racialized and gendered nature of the workforce for essential frontline workers. Disproportionate impacts of precarious work on women are recognized by the United Nations which states that:

“not enough attention is given to how their [women] work environment may be discriminatory, as well as what their sexual and reproductive health and psychosocial needs are as frontline health workers” [25] .^page 1^

During the pandemic, women’s domestic burden has also increased. In general, most of the caregiving at home is provided by women. With the pandemic, unpaid caregiving work by women and girls has increased [26]. For example, taking care of children who are at home due to school closures disproportionally impacts women [24]. Women are also the main caregivers of those infected by the virus within the household, increasing their unpaid housework and also putting them at higher risk [25]. Outside the home, workplace violence has increased against female health care workers in China, Italy, and Singapore [27]; and in the USA, there have been reports of increased violence directed at street-based sex workers [27]. Victims of sex trafficking are among the most vulnerable and marginalized groups at risk and this can affect underage girls as well as women [28, 29].

### Gender-Based Violence Risk Factors During a Pandemic

Structural and systemic factors in the context of the COVID-19 pandemic impact violence against women and girls [30]. Poverty, under/unemployment, informal precarious work, and overall financial challenges result in increased household stress, and higher rates of exploitative, transactional sex among girls. Women with precarious immigration status or non-status are at increased risk. Factors that contribute to increased risks for immigrants include stress associated with migration, change in social networks and supports, change in gender roles and responsibilities, and economic insecurity [31]. Women with precarious status are excluded from government relief programs because access to such programs is dependent on legal residency status [9]. Immigrant and refugee women and girls may face specific barriers to reporting GBV and seeking help including, for example, fear of loss of children due to deportation, limited knowledge about their rights, and services available, and discrimination and racism.

Social distancing, reduced community interactions, restriction of movement, and closure of services limit women’s ability to distance from their abusers and to access the supports they need [5]. This is of particular concern for marginalized groups like Indigenous Peoples who worldwide face increased risks of family violence (for example, Native American women face victimization at rates that are two and a half higher than Caucasians; and in Canada Indigenous women experience it two and a half times than non-Indigenous women) [32]. Previous emergencies have shown that women and other marginalized groups face reduced access to services, for example, in relation to abortion, HIV, and mental health services. Reduction and diversion of resources for essential services impact women’s sexual and reproductive health. In past crises, this resulted in “increased rates and sequelae from unintended pregnancies, unsafe abortions, sexually transmitted infections… post­traumatic stress disorder, depression, suicide, intimate partner violence, and maternal and infant mortality” [33].^page1176^ During the Ebola outbreak in Sierra Leone, more women died of obstetric complications than of the virus itself [33]. The UNICEF [24] reported that life-saving care and support services for gender-based violence survivors, including clinical management of rape, mental health services, and psycho-social support, are disrupted in tertiary level hospitals given that health service providers are overburdened and preoccupied with handling COVID-19 cases. Hall et al. [33] note that international responses to COVID-19 perpetuate a disregard for sexual and reproductive health and further contribute to justice inequities that impact negatively on the wellbeing, health, and economic stability of women and vulnerable populations in general.

The closure of schools as part of COVID-19-related measures has further disrupted girls’ education. A study on the gendered effects of school closures during the COVID-19 pandemic reports teenage girls in developing countries may disproportionately drop out of school due to increased risk of pregnancy, forced marriage, and sexual exploitation [34]. The burden of unpaid household work has also disproportionately affected girls. Girls between 5 and 14 years are spending 40% more time than boys doing unpaid work at home; as a result, girls may spend less time studying or may drop out of school [34].

Pandemics like COVID-19 exacerbate not only violence within the home, but other forms of violence against women occurring outside of the home. Scholars refer to the concept of structural violence to encompass the “often-hidden ways” that poverty, racism, discrimination, and other systems of inequality (such as health) impact the lives of marginalized populations [14]. For example, racism and xenophobia in the workplace have also been reported in the context of the current COVID-19 pandemic. In a recent survey conducted with health care workers in Manitoba, Canada, it was found that 1 in 5 workers who identified as Asian experienced racism in the workplace [1, 35]. Increase in workplace violence towards female health care workers during the pandemic has been reported in China, Italy, and Singapore [27]. Sinlcair and colleagues [36] recommend deploying mental health support to racial minority workers, in particular Asian workers, because of the micro- and macro-aggressions they likely experience during the pandemic.

### Racism, Discrimination, and Health Outcomes

Racialized communities bear a disproportionate burden of stress, illness, and health inequities, and *racism* is a systemic risk factor. In the USA, alarming rates of COVID-19 infections and deaths among Black Americans, and overall disproportionate impacts on racialized people from ethnic minority backgrounds, illustrate health inequalities [22]. Racialized populations, including Indigenous peoples across the world, are the hardest hit in this pandemic; where their social determinants of health are jeopardized by poverty, inequitable access to medical care or health advice, inadequate housing, precarious employment, and immigration status [32, 37, 38]. Reports show the death rate from COVID-19 among African Americans is 2.4 times the death rate of White Americans in the USA [39]. Similar findings have been reported in Toronto, Canada; the pandemic has had a greater impact on Black and other racialized populations. Black and other racialized groups constitute nearly half of Toronto’s population but represented 83% of reported COVID-19 cases [40]. Furthermore, Black individuals make up 9% of the city’s population, yet they represented 21% of the reported cases [40].

Overcrowded and low-income households are disproportionately impacted by the pandemic [40]. Experiencing health disparities, as a result of the socioeconomic circumstances, stress related to racism and discrimination, and work requiring close contact with others, prevents following public health responses to COVID-19 [40]. It is in this context that researchers and advocates have highlighted the need to address the lack [41] or inconsistency [42] of data collection in relation to key indicators, such as race, ethnic, gender, age, ability, sexual identity, to fully understand the impacts of the pandemic on marginalized groups.

The concomitant effects of the COVID-19 pandemic and violence against women amplify health inequities. Furthermore, systemic racism with its accompanying discrimination and prejudice contributes to barriers in accessing sexual and reproductive health care for racialized women. In North America, historic institutional mistreatment, racism and discrimination against Indigenous communities, and lack of services run by Indigenous people are among the barriers to women and girls seeking survivors’ services [35]. Disparities in access and availability to health and mental health care services increase the vulnerabilities of women and girls [9]. In the Canadian context, there have been high rates of reported homicide and suicide among Indigenous communities, which also put women and girls and people from other sexual orientation at higher risk [9]. For example, between 2011 and 2016, statistics show that suicide rates were 3 times higher among First Nations people than among non-Indigenous people; disparities between the two groups were wider among age groups under 15 years, and especially among females between 15 and 24 years of age [43]. Housing shortage, substandard housing, and lack of safe houses and shelters on reserves increase the risk to violence faced by First Nations women, girls, and children [38]. Long-standing inequities in the funding of child welfare services, along with COVID-19 social isolation measures, increase the risk faced by First Nations women, girls, and children, who are disproportionately affected by physical, domestic, and sexual violence [38]. Issues of trust in public institutions and agencies providing services also create barriers to accessing services for survivors, impacting them negatively [35].

Xenophobia and racism during the pandemic have increased. In Canada, racialized individuals were three times more likely than the rest of the population to perceive increases in the frequency of harassment, attacks based on race, ethnicity, or skin color. In particular, 1 in 3 racialized women felt unsafe compared with racialized men (1 in 5) [44]. Racist stereotypes against individuals from Asian background have also resurfaced during the current COVID-19 pandemic; in less than a month, a total of more than 1700 anti-Asian hate incidents were documented in the USA [45].

In light of this setting, it is not surprising that the mental health impacts of COVID-19, the “fourth pandemic,” will be the biggest aftermath of the pandemic and the greatest impact of the health footprint will be borne by marginalized and racialized populations [46]. Mental health experts predict acute rise in psychiatric disorders and a “tsunami” of mental health issues, and higher risks of poor psychological wellbeing among marginalized populations [47]. Among them are women, low-income families, individuals with limited social support network, and those experiencing social isolation, economic stress, stigma, racism, and social exclusion. For example, a survey in Canada illustrates that during the pandemic Indigenous women are experiencing higher rates of violence and are, therefore, more concerned about intimate violence than COVID-19-related issues (such as financial) [48]. Indigenous Peoples were also more likely to have suicidal thoughts (16% vs 6% for others), and feel depressed (31% vs 23% for others) [49]; and fear of domestic violence was reported twice as likely among racialized people and high among Indigenous Peoples [46] (Table 2).

**Table 2:** Characteristics of selected included literature [20]

Selected peer-reviewed articles			
No	Author	Type of document/Title	Focus/purpose/objectives/goal
1	Burzynska and Contreras (2020)	Article. Gendered effects of school closures during the COVID-19 pandemic	Vulnerability of girls in the COVID-19 pandemic
2	Chai (2020)	Book chapter	Spread of anti-Asian racism: prevention and critical race analysis in pandemic planning
3	Craft, McGregor, and Hewitt (2020)	Book chapter	First Nations’ responses to COVID-19
4	Day and White (2020)	Article. Gender-specific online content is important and timely for women receiving treatment for substance use disorders	Gender-specific interventions for women seeking drug and alcohol treatment
5	Hall et al. (2020)	Commentary. Centering sexual and reproductive health and justice in the global COVID-19 response	Intersectional approaches and community-driven interventions to tackle gendered disparities during COVID 19
6	John et al. (2020)	Article. Lessons never learned: crisis and gender-based violence	Use experiences from previous emergencies as lessons to address GBV during the COVID-19 pandemic
7	Knaul et al. (2019)	Comment. Countering the pandemic of gender-based violence and maltreatment of young people: The Lancet Commission	Knowledge creation to counter the pandemic. The Lancet Commission on GBV and Maltreatment of Young People intend on producing new tools and data
8	Levesque and Thériault (2020)	Book chapter	Systemic discrimination in government services on First Nations Peoples in the COVID-19 pandemic context
9	Roesch et al. (2020)	Editorial. Violence against women during covid-19 pandemic restrictions	Gendered effects of the COVID-19 pandemic
10	Souza et al. (2020)	Article. Violence against women, children, and adolescents during the COVID-19 pandemic: overview, contributing factors, and mitigating measures	Effects of social distancing on interpersonal relations, such as intimate partners and between parents and children
11	Van Gelder (2020)	Comment. COVID-19:Reducing the risk of infection might increase the risk of intimate partner violence	Effects of the COVID-19 pandemic in the rise of IPV
Selected grey literature			
No	Author/institution	Type of document	Focus/objectives
1	Abji, Pintin-Perez, and Bhuyan (2020)	News article	Impacts of COVID-19 on non-status women
2	Astrup 2020)	News article	Explores the worrying surge in domestic abuse during the COVID-19 lockdown and what is being done to address it
3	Bielski (2020)	Newspaper article	Provides recommendations to address intimate partner violence during the COVID-19 pandemic
4	City of Toronto (2020)	Report	COVID-19: status of cases in Toronto
5	Cortez (2020)	Congressional documents and publications	Letter led by U.S. Senator Bob Casey urging Senate leadership and appropriators to support emergency funding to the Department of Health and Human Services for family and domestic violence programs
6	CREVAWC (2020a)	Infographic	Contributing factors to risk and vulnerabilities associated with intimate partner violence in immigrant and refugee communities
7	CREVAWC (2020b)	Infographic	Identifies the barriers to reporting and disclosing violence and seeking help
8	CREVAWC et al. (2020)	Publication	Cross-cutting risks and GBV recommendations in Canada, focus on 2SLGBTQ + , Indigenous, Black, and ethnic minority communities
9	EIGE (2020)	Website	The gendered issue of COVID-19. Makes suggestions to policymakers
10	Enekwechi, Hardeman, and Powell (2020)	Webinar	Health inequities, social determinants of health in the context of COVID-19—Black, Indigenous, and racialized population (Latinx, American Indian, Alaskan Native)
11	Fraser (2020)	Research report	Review of literature. evidence on the impact of the COVID-19 virus pandemic and other similar epidemics on violence against women and girls
12	Gopal and Adesara (020)	Blog	Addresses racism and health inequalities
13	Ho (2020)	Newspaper article	Enforcement of COVID-19 measures is disproportionately impacting Black, Indigenous, and marginalized communities
14	IPU (2020)	Guidance note	Provides guidance to parliaments around the world in relation to COVID and gender
15	Junker (2020)	News article	Explores the different ways the pandemic may hide domestic violence and probable strategies and responses to help women
16	NCLW et al. (2020)	Issue alert	Provides updates on gender issues in Lebanon, complies data to inform programs, and offers recommendations to combat gender issues related to GBV
17	OCASI (2020)	Report	Environmental Scan – Building Leadership Capacity to Address Gender-Based Violence against Non-Status, Refugee, and Immigrant Women Across Canada
18	O’Donnell, Peterman, and Potts (2020)	Blog	Pathways linking pandemics and violence against women and children
19	Onyango (2020)	Blog	Draws lessons from Ebola that apply to the COVID-19 pandemic
20	Rezaee (2020)	Article	Women, girls, and gender-diverse communities’ experiences to help formulate a clear understandings of COVID-19 impacts
21	Roush (2020)	Blog	Critically analyzes the “invisible” danger of pandemic “safety” measures
22	Senior (2019)	Blog	Racialized mothers’ specific needs
23	Siangyen (2020)	Blog	Addresses gender inequalities in Asia’s response to COVID-19
24	UNDP (2020)	UNDP brief	GBV during COVID
25	UNICEF (2020)	Technical note	Recommendations for gender equality in the COVID-19 response
26	UNFPA (2020a)	Interim technical brief	To advocate for the rights of women and girls during the COVID-19 pandemic
27	UNFPA (2020b)	Technical brief	Providing recommendations for pandemic responses with a specific focus on pandemic effects on women and girls
28	UN Women (2020a)	Brief	Emerging evidence on the effect of the pandemic on GBV and recommendations
29	UN Women (2020b)	Brief	Reveals a trend in violence against women and children in public spaces, provides recommendations
30	UN Women (2020c)	Brief	Trends and implications of providing essential services for survivors
31	UN Women (2020d)	Complementary note	Summarizes data collection principles and recommendations
32	WHO (2020)	Website	Outlines an informative plan with suggestions of steps to take when experiencing violence at home
33	Women Services Network (2020)	Toolkit	Best practices for frontline workers on service provision for victims of abuse through mobile delivery
34	Yang et al. (2020)	Newspaper article	Public health report on impacts of COVID-19 in Toronto

## Discussion: Public Health Implications

**Fig. 2:**
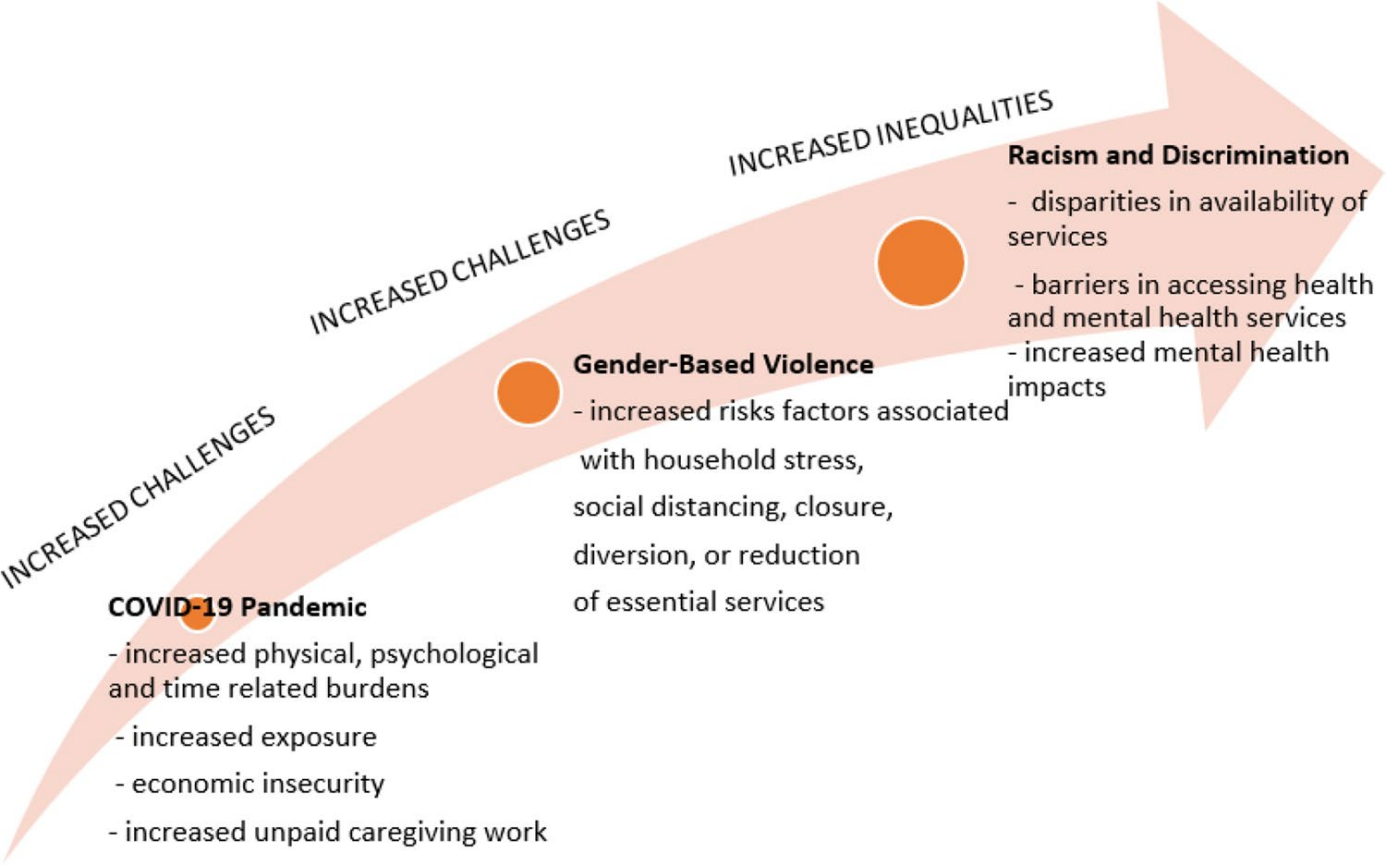
2020 Syndemic: convergence of COVID-19 pandemic, gender-based violence, and racism

We applied an intersectionality perspective to understand how gender intersects with the social determinants of health, leading to gendered and racialized pathways in health disparities, and resulting in synergistic health disadvantage. This disparity is higher among certain segments of the population, including Indigenous, Black, and other racialized women at risk of gender-based violence during COVID-19 pandemic’s response and recovery phases (Fig. 2). The pandemic’s disproportionate risks and impacts bring into light historic, systemic, and structural inequalities at the intersection of racial and ethnic minority status, occupation, and class. As Horton notes, a purely biomedical solution to tackling COVID-19 will likely fail, no matter how effective COVID-19 vaccines and treatments may be because the truth that lies behind these vulnerabilities is barely admitted (2020, p. 874).

Informed by our synthesis review, in Table 3 we highlight public health and mental health implications. International organizations and advocates have recommended that to promote gender equality, and address violence and discrimination, it is imperative to integrate *gender-responsive programming* into countries’ strategic plans for COVID-19 preparedness and response [26, 50]. Scholars applying a syndemic approach have pointed out the political, economic, and social intersecting factors that, in the context of power inequalities, increased the vulnerabilities and susceptibilities of women to health risks like HIV/AIDS [51]. Our review underscores that COVID-19 response strategies should take into account gendered roles and dynamics, power relations, and responsibilities [24]. By integrating a gender-specific lens, we may be able to consider invisible factors, such as the burden of paid and unpaid care work for women during the health emergency and GBV risks, in planning and response programming [24]. Applying a gender-specific lens we may be able to oversee governments’ responses to the health emergency by asking: whether these interventions ensure that women and girls have access to protection, resources, and shelters as essential services; what is being done to curb the impact of the outbreak on support services for survivors, particularly health care, police, and justice services; how specific actions to protect women survivors of violence have been adapted and included in the emergency measures; about the codes of conduct in place to address the endemic violence against female health workers and sexual harassment in the health and social sectors; and about measures to protect girls at risk of sexual violence in the context of schools closures [52].

**Table 3:** Selected recommendations with public health and mental health implications

Recommendations	Implications
Gender-responsive COVID-19 programming	✓ Consider GBV a public health issue ✓ Increased dedicated funding for survivor services and supports ✓ Ensure women and girls have access to protections during crises ✓ Implement codes of conduct in place to address the endemic violence against female health workers and sexual harassment in the health sector ✓ Engage in multisectoral strategic responses—health, housing
Intersectional and critical race lenses to emergency health responses	✓ Include diversity of voices and perspectives from Black and Indigenous communities, and other racialized population, to ensure equity and comprehensive pandemic and post-pandemic responses ✓ Recognize the existence of differentiated primary and secondary effects of the health emergency on marginalized people ✓ Apply anti-racism, anti-oppression, and equity policy to service provision
Disaggregated data collection	✓ Collect disaggregated data—race, gender, sex, ethnicity, age, disability, occupation, socioeconomic status, migratory status, geopolitical location
Mental health	✓ Expand access points to mental health services ✓ Conduct mental health audits and inequality impact assessments
GBV referral	✓ Update referral pathways and promote institutional multisectoral collaborations

Abbreviations: *GBV*, gender-based violence

Mendenhall [53] argues that framing the current health emergency as a global syndemic is misleading because it overlooks the primary factors that mobilize clustering and disease interaction. Applying a syndemic perspective, Mendenhall [53] observes, allows the integration of the historical legacy of systemic racism, which explains the factors accounting for why and how the coronavirus is moving through the population in the USA, and the interaction with social and biological factors. Critical health researchers recommend application of critical *race and intersectional analysis* to the study and evaluation of public health response [54]. Health authorities should ensure non-discriminatory access to health care services [47]. Furthermore, to decolonize Indigenous health implies the design of policies that apply Indigenous social determinants of health model [55]. A decolonizing approach to health promotion will address immediate needs as well as structural causes of Aboriginal health inequities [56]. There is also a need to address systemic and micro-racist, microaggression practices in health care provision [57]. There is a need to decolonize care, in other words, to provide racism-free care in the health care system [57].

Gravlee [12] argues that the framework of the syndemics is useful to apply to prevent the danger of reframing the phenomena of Black people being disproportionately impacted by COVID-19, as part of a “intrinsic Black vulnerability.” These interpretations, Gravlee explains [12], have emerged and respond to the legacy of racial-genetic discourses in American medicine. Fraser’s [27] literature review considered how the current coronavirus pandemic may impact on violence against women and girls. She found that there is limited data on how levels of violence change and are transformed during emergencies, and about the pathways of violence during crises. Data collection is a strategic tool for understanding how and why emergencies such as the COVID-19 pandemic increase violence against women and girls around the world [58]. To address the specific needs of racialized and marginalized communities, institutions should gather *disaggregated data* including gender, age, sex, race, ethnicity, disability, occupation, socioeconomic status, migratory status, and geographic location [25, 41, 42]. The collection of disaggregated data is also key in planning equitable responses to the pandemic, and in addressing the needs of diverse communities during its different waves and recovery phases. As highlighted by Indigenous scholars, “failure to recognize the differences in morbidity and mortality among Indigenous Peoples contributes to inequities” [32].^page 2739^.

*Mental health* access points should be promoted to address mental health gaps in communities at greater risks and with higher disparities [1]. It is recommended to conduct mental health audits and inequality impact assessments of COVID-19 pandemic and post-pandemic policies across all sectors [47]. Furthermore, early in the pandemic we made calls for a public mental health system, to transform our approach to mental health and wellbeing across sectors [59].

*GBV referral pathways* should be updated to reflect care facilities’ availability, and mechanisms to inform communities and service providers [25]. Updates in service directories are needed, and dissemination of this information regularly among strategic networks should be conducted. Collaborations between the health sector and organizations involved in anti-GBV initiatives are needed during emergencies, to provide services effectively and to strengthen referral pathways in accordance with COVID-19 mitigation measures. Updates to referral pathways are important to prevent overwhelming tertiary hospitals [27].

## Limitations

Our review has several of limitations which we acknowledge. First, the peer-reviewed and grey literature on COVID-19 is rapidly growing. Through our search we may have missed more recent peer-reviewed publications, or pertinent grey literature that are not posted on publicly available websites or other media. Second, we applied a particular analytical lens to interpreting the findings. While we believe that an intersectionality lens is the most pertinent in addressing the synergistic impact of GBV and racism on women during the COVID-19 pandemic, other researchers may arrive at other interpretations, depending on their theoretical and analytical lens. Third, due to word limitation in journal submission, we did not elaborate on all the literature that we located as part of our synthesis review. We invite interested readers to access our publicly available rapid synthesis findings at https://cihr-irsc.gc.ca/e/52062.html. Finally, our review methodology followed a particular rapid synthesis approach. We did not engage in a full scoping review or a systematic review. However, we believe the Cochrane Rapid Reviews method is well recognized, with detailed steps that allow for repeat of a review for verification purposes by other researchers.

## Conclusion

The experiences of racialized women highlight the differentiated risks, marginalization, social injustices, and inequalities they face, which have been always present, but have compounded in the context of the current COVID-19 pandemic. Drawing from a rapid synthesis of literature, we argue that racialized women are experiencing a *2020 Syndemic*: a convergence of COVID-19, gender-based violence, and racism pandemics, placing their wellbeing at a disproportionate risk. We underscore the need to collect disaggregated data and provide recommendations taking into consideration the particular social determinants of mental health of racialized women and girls who have experienced violence during the pandemic.

## Data Availability

Not applicable.
